# What would be the impact of COVID-19 on liver function of a patient with chronic hepatitis B? About a case and literature review

**DOI:** 10.11604/pamj.2021.38.225.28123

**Published:** 2021-03-01

**Authors:** Khoury M'bodj, Hakima Abid, Najdi Adil, Mohammed El Abkari, Nourdin Aqodad

**Affiliations:** 1 Faculty of Medicine and Pharmacy of Fez, Sidi Mohamed Ben Abdellah University, Laboratory of Human Pathology, Biomedicine and Environment, Fez, Morocco,; 2 Faculty of Medicine and Pharmacy of Fez, Sidi Mohamed Ben Abdellah University, Hassan II University Hospital Center, Fez, Morocco,; 3 Faculty of Medicine and Pharmacy of Tangier, Abdelmalek Essaâdi University, Tangier, Morocco,; 4 Faculty of Medicine and Pharmacy of Agadir, IBN ZOHR University, Hassan II Hospital, Agadir, Morocco

**Keywords:** SARS-CoV-2, hepatitis B, liver function, case report

## Abstract

Liver damage during COVID-19 disease has been described in numerous studies. Its mechanism is poorly understood. It is mainly reserved for severe forms and is manifested by abnormalities of the hepatic assessment and more particularly cytolysis. Particular attention must be paid to patients with chronic liver disease, both in terms of follow-up and treatment. We wanted to know the evolution of COVID-19 and its treatment, on the liver function of a 27-year-old patient followed for chronic non-cirrhotic hepatitis B at the Hassan II University Hospital in Fez. Our patient had stopped the antiviral B treatment and presented COVID-19 infection with minimal to moderate impairment. The initial evaluation showed cytolysis at 4 times upper limit of normal (ULN). Management consisted in the immediate resumption of Tenofovir in combination with hydroxychloroquine (HCQ) and azythromycin with good clinical and biological evolution.

## Introduction

SARS-CoV-2 is responsible of coronavirus 2019 (COVID-19) [1]. According to the WHO report of 23/09/2020, there are 31,425,029 confirmed cases of COVID-19 and 967,164 deaths worldwide. In Morocco, 105346 cases have been confirmed and the number of deaths has been estimated at 1889 [2]. COVID-19 may be asymptomatic or manifest itself as headache, sore throat, cough, fever, fatigue, myalgia, dyspnea and conjunctivitis. It can progress to acute respiratory distress syndrome (ARDS) followed by death [1]. A recent study found that 2-11% of COVID-19 patients had a history of chronic liver disease, with 14-53% of patients developing COVID-19 related liver damage [3]. These liver lesions are associated with severe disease progression or mortality [4]. Chronic hepatitis B virus (HBV) remains a public health problem with 257 million people affected worldwide [5]. The study by Chen *et al*. reported that patients with COVID-19 in addition to HBV tend to develop severe liver damage and progress to a critical phase of the disease [4]. In Morocco, as in many countries, HCQ, which is an immunosuppressive treatment, and azithromycin, which is an antibiotic, have been used in the treatment of COVID-19. However, researchers reported that HCQ was independently associated with hospital mortality in COVID-19 patients and azithromycin had a negative impact on transaminases [6,7]. In view of this information, we wanted to know the evolution of COVID-19 and its treatment on the hepatic function of a patient who was a chronic carrier of HBV.

## Patient and observation

The patient was a 27 year old man, followed at Hassan II University Hospital of Fez for chronic hepatitis B discovered during an asthenia check-up in 2014. He had a level of alanine aminotransferase (ALT) equal to 1.26 times ULN and ULN aspartate aminotransferase (AST). The HBs Antigen was positive, Antigene HBe positive with HBe Ac negative and a viral load of 45144142 IU/ml (8.06 log IU/ml). The blood test was normal, hemoglobin=18.10dL, leukocyte=5.12*103μL, poly neutrophils=1.62*103μL, lympocytes=2.85*103μL, platelet=218*103μL, serological test for hepatitis (hepatitis C, Hepatitis D), HIV and sypilica was negative. Liver ultrasonography was normal and the fibroscan showed an elasticity of 5.6 Kpa. For reasons of financial means, the patient was out of sight. In 2017, he reconsulted with cytolysis, ALT=5.74 times ULN, AST=2.06 times ULN, a viral load of 6.63 log IU/ml. The fibroscan was redone with an elasticity measured at 10.8 Kpa. Thus a liver biopsy was performed, giving a Metavir score of A1F2. An antiviral treatment based on tenofovir was prescribed but was only started in January 2019 with an improvement of the hepatic assessment and a viral load at S24 at 260 UI/ml. In April 2020, he presented with a SARS-CoV-2 infection and reported that he was no longer on antiviral B therapy. The patient had presented with influenza-like illness with headache, myalgia and fever of 38.3°C. His liver workup was disturbed with ALT at 4.4 times ULN, AST at 2.04 times ULN, GGT at 1.4 times ULN. Cardiac, respiratory and blood counts were normal. Chest CT scan showed 2 nodules in the right upper lobe of frosted glass with minimal damage estimated at <10%. Management consisted of emergency resumption of Tenofovir in combination with low-dose azythomycin and HCQ. At d14 of his hospitalization. At d18, the ALT and AST levels were 6 times ULN and 2.74 times ULN respectively. At d21, his CT scan showed a total regression of the lesions. He was hospitalized for 35 days. At discharge, the patient's liver function tests improved with a decrease in transaminase levels from baseline from 4.4 times ULN to 3.66 times ULN and from 2 times ULN to 1.44 times ULN for ALT and AST respectively. In July 2020, the patient had a normal liver function test with a negative viral load.

## Discussion

Data on the exact prevalence of viral hepatitis in COVID-19 patients are limited, in a meta-analysis, the prevalence of underlying chronic liver disease was estimated at 3% and was significantly associated with severe disease progression and mortality, it should be noted that most of these studies were conducted in China where the prevalence of HBV is high [8]. Hepatic dysfunction is observed in 14 to 53% of COVID-19 patients [3]. The etiology of hepatic injury in COVID-19 patients is multifactorial. It may result from a cytopathic effect of the SARS-CoV-2 virus, the effect of COVID-19 drugs or an underlying liver disease [9]. Patients carrying a hepatitis virus have transiently elevated transaminases that may result from activation of the immune system induced by the virus in question [10]. Likewise, patients who stop their antiviral treatment (e.g. Tenofovir) usually have a virological and biochemical relapse leading to a reactivation of HBV and a worsening of clinical cases. In our case, the patient responded favorably to antiviral therapy and discontinued it, which could be a cause of transaminase exacerbation [11]. The entry of SARS-CoV-2 into the body is via the ACE2 receptors. These receptors are abundantly expressed in type 2 alveolar cells but also in 59.7% cholangiocytes in 2.7% hepatocytes, making the liver a target organ of the coronavirus [9]. Documented sources have reported that COVID-19 is associated with abnormal liver enzyme levels [12]. This could be consistent with our patient's case. He had elevated transaminases on admission.

The Wang *et al*. study demonstrated that SARS-CoV-2 had a direct effect on the liver, based on the results of a post mortem biopsy in 2 patients of COVID-19, the patients had elevated transaminases, SARS-CoV-2 particles were identified in the cytoplasm of hepatocytes, mitochondria were swollen, endoplasmic reticulums were dilated and glycogen granules were decreased [13]. This proves that SARS-CoV-2 is not only able to enter but also to replicate in hepatocytes. A similar study suggested that SARS-CoV-2 could induce cellular apoptosis leading to liver damage. From another point of view, azitromycin is one of the macrolides, which are antibiotics that are toxic to the liver and imperatively in cases of decompensated cirrhosis [14]. During his hospital stay, our patient had a higher transaminase level than when he was admitted, which may be related to an over-added drug origin. HCQ, azithromycin or the HCQ + azithromycin combination is one of the therapeutic alternatives that can eliminate SARS-CoV-2 [15]. In vitro studies have shown that HCQ or the combination of HCQ and azithromycin would inhibit SARS-CoV-2 [16]. A similar study noted that the combination of HCQ and azithromycin had a synergistic effect and would allow clearance of SARS-CoV-2. Chinese studies found that the excretion of COVID-19 virus ranged from 19 to 37 days [17], which is consistent with our patient's case. Patients with HBV treated with immunosuppressive drugs are at risk of reactivation of HBV and therefore morbidity and mortality. Helbling *et al*. reported cases of viral reactivation after treatment with chloroquine [18].

In the study by Liu *et al*. two patients treated with glucocorticoids (immunosuppressants) to eliminate SARS-CoV-2, also had viral reactivation [5]. Therefore, it is imperative to ensure that the patient continues antiviral B therapy after stopping HCQ and to perform regular transaminase testing. Drug interaction is unlikely between Tenofovir, HCQ and azithromycin because they have different pathways of metabolism and clearance [19]. Zhang *et al*. found that the course of COVID-19 patients and those with over-infected HBV were not significantly different [20]. Thus, a SARS-CoV-2 and HBV co-infection has no effect on the length of hospitalization, the patient's prognosis or the severe progression of the disease. Our patient progressed well during hospitalization despite persistent cytolysis. Unfortunately, we are not able to determine the exact origin of the liver abnormality. Further studies are needed to evaluate the impact of SARS-CoV-2 on HBV.

## Conclusion

Liver damage during SARS-CoV-2 infection is common, especially in severe forms or chronic liver disease. In our patient with chronic HBV, poor compliance with therapy is added to the possible causes of liver dysfunction in the case of this association. Moreover, the evolution was good under treatment with emergency resumption of the antiviral B. We recommend careful monitoring of biochemical parameters.
